# *Mycobacterium tuberculosis* Rv0927c inhibits the proliferation and promotes the intrinsic apoptosis of alveolar epithelial cells through targeting mitochondrial TUFM

**DOI:** 10.3389/fimmu.2026.1720489

**Published:** 2026-03-18

**Authors:** Aihong Xia, Xin Li, Xiang Chen, Juanjuan Quan, Jiaxu Wan, Chengkun Zheng, Zhengzhong Xu, Xinan Jiao

**Affiliations:** 1 Jiangsu Key Laboratory of Zoonosis/Jiangsu Co-Innovation Center for Prevention and Control of Important Animal Infectious Diseases and Zoonoses, Yangzhou University, Yangzhou, China; 2 Key Laboratory of Prevention and Control of Biological Hazard Factors (Animal Origin) for Agrifood Safety and Quality, Ministry Of Agriculture and Rural Affairs, Yangzhou University, Yangzhou, China

**Keywords:** apoptosis, lung epithelial cells, Mycobacterium tuberculosis, proliferation, Rv0927c

## Abstract

**Introduction:**

*Mycobacterium tuberculosis (M. tuberculosis)* is the causative agent of tuberculosis (TB), which continues to be a leading cause of death from infectious diseases globally. Lung epithelial cells play a crucial role in the infection process of *M. tuberculosis*. However, the specific *M. tuberculosis* proteins that regulate lung epithelial cells remain to be identified, and the mechanisms underlying the interaction between *M. tuberculosis* and lung epithelial cells are still not fully understood.

**Methods:**

In this study, CCK8 assay and xenograft tumor models were employed to investigate the effect of *M. tuberculosis* protein Rv0927c on the proliferation of lung epithelial cells (line A549). Flow cytometry was used to detect cell apoptosis, Western blot analysis was performed to examine the cleavage levels of apoptosis-related proteins, and JC-1 staining assay was conducted to assess mitochondrial membrane potential. Additionally, the interaction between Rv0927c and host TUFM molecules was verified by immunoprecipitation, and the role of this interaction in Rv0927c-induced apoptosis was also explored.

**Results:**

The results showed that Rv0927c inhibits the proliferation of lung epithelial cells both *in vitro* and *in vivo*. Flow cytometry analysis demonstrated that Rv0927c significantly increased apoptosis in A549 cells. Additionally, Rv0927c facilitated the cleavage of caspase-3, caspase-9, and PARP, while having no effect on the cleavage level of caspase-8, and it led to a decrease in mitochondrial membrane potential. Furthermore, Rv0927c interacts with host TUFM molecules, which is necessary for Rv0927c to promote apoptosis in host cells.

**Discussion:**

Our findings provide evidence that Rv0927c inhibits proliferation and regulates apoptosis by targeting TUFM in A549 cells, which contributes to the understanding of the mechanisms underlying the interaction between *M. tuberculosis* and lung epithelial cells.

## Introduction

Epithelial cells in the lungs, located at the interface between the external environment and the interior body, can be categorized into two main types: airway epithelial cells and alveolar epithelial cells (1). Airway epithelial cells, serve as a physical and functional barrier against pathogen invasion (2), which can produce antimicrobial factors, promote inflammatory responses, and regulate gas exchange (3, 4). Alveolar epithelial cells include type I alveolar epithelial cells (AEC I) and type II alveolar epithelial cells (AEC II). Both types appear to participate in *M. tuberculosis* infection, but AEC II have greater immunological activity. AEC-II secrete cytokines and chemokines, participate in immune cell activation and differentiation, and express MHC class II molecules (5–9). Alveolar epithelial cells, particularly AEC II cells, appear to play a dual role during *M. tuberculosis* infection. It was previously thought that *M. tuberculosis* evades macrophage-mediated killing by infecting epithelial cells, which are non-professional phagocytes, thereby creating a favorable ecological environment for bacterial replication and the establishment of infection (10, 11). However, a growing body of evidence suggests that AEC II cells are important for fighting tuberculosis. AEC II cells can secrete a variety of surfactant proteins, including SP-A, SP-B, SP-C, and SP-D (12). As part of the lectin family, SP-A and SP-D function as immune molecules that contribute to bacterial clearance (13). García-Pérez et al. found that mycobacteria enters epithelial cells through macropinocytosis and is killed by A549 cells (14). Further studies revealed that epithelial cells initiate the anti-mycobacterial inflammatory response by producing IL-8, MCP-1, and IFN-γ (15). Additionally, alveolar epithelial cells recognize *M. tuberculosis* to trigger the host innate immune response through pattern recognition receptors (16) and presents *M. tuberculosis* antigens to CD4 T cells (5), thereby initiating the adaptive immune response.

In mouse models, chronic tuberculosis infection has been shown to induce lung cancer (17). Additionally, BCG has been found to protect A549 and several other tumor cells from TNFα-induced apoptosis, thereby promoting tumorigenesis in xenograft model (18). These effects are closely related to the regulation of cell proliferation. Furthermore, *M. tuberculosis* effector proteins Mce2E and PtpA have also been implicated in the promotion of A549 proliferation and tumor formation (19, 20). However, numerous *M. tuberculosis* proteins that regulate lung epithelial cells proliferation remain to be identified in *M. tuberculosis* (totaling 4093 genes encoding 3993 proteins).

Host cells can resist *M. tuberculosis* infection through apoptosis. Apoptotic cells were ingested by macrophages, leading to the elimination of intracellular bacteria (21). However, apoptotic cells may also facilitate the escape of bacteria to neighboring cells and then to other tissues, spreading infection (22). Multiple *M. tuberculosis* proteins are now known to regulate macrophage apoptosis, including the 19-kDa glycolipoprotein (Rv3763) (23), PE_PGRS33 (Rv1818c) (24), ESAT6 (Rv3875) (25), and the 38-kDa lipoprotein (Rv0934) (26). Nevertheless, research concerning the regulation of lung epithelial cell apoptosis by *M. tuberculosis* proteins remains in the early stages.

The *M. tuberculosis* gene Rv0927c encodes a short dehydrogenase/reductase and is associated with the synthesis of mycotic acid in the cell wall of mycobacteria (27). Previously, we demonstrated that Rv0927c modulate the host innate immune response and promoted bacterial survival in macrophages (28). To investigate the potential function of Rv0927c in lung epithelial cell, we used *in vitro* and *in vivo* proliferation models to assess the effect of Rv0927c on host cell proliferation. Additionally, we evaluated the ability of Rv0927c to induce host cell apoptosis and explored the underlying molecular mechanisms. Our findings suggest that Rv0927c may function as a regulator of lung epithelial cell proliferation and induce apoptosis in a TUFM-dependent manner.

## Materials and methods

### Cells

HEK293T cells (InvivoGen, San Diego, CA, USA) and lung epithelial (A549) cells were cultured in complete DMEM (Gibco, Grand Island, NY, USA) containing 10% fetal bovine serum (GIBCO), 100 U/mL streptomycin, and 100 U/mL penicillin (GIBCO) at 37°C and 5% CO_2_.

### Cell count assay

The CCK8 assay was conducted using a Cell Counting Kit-8 kit (FcMACS, Nanjing, China). Briefly, A549 cells were seeded into 24-well plates at a density of 5×10^4^ cells/well and cultured overnight. They were then transfected with pCMV-Myc (500 ng) or pCMV-Myc-*Rv0927c* (500 ng) using Lipofectamin^®^ 3000. At 0, 24, 48, and 72 h post-transfection, cell supernatants were discarded, and CCK8 working solution was added. The plate was then incubated at 37 °C for 1 h before optical density (OD) at 450 nm was measured using a microplate reader.

### Retroviral vector construction and retrovirus packaging

Full-length Rv0927c gene was amplified from *M. tuberculosis* H37Rv genomic DNA by PCR using existing primers (F: 5′-GCCGGAATTAGATCTCTCGAGATGATCCTGGATATGTTCCGTCTT-3′; R: 5′-CTCCCCTACCCGGTAGAATTCTCAGTGATGATGGTGATGATGCAGGTCCGGAATGGGA-3′). A tag containing six histidine residues was included in the *Rv0927c* C-terminus. Amplicons were digested and ligated into the pMSCVpuro retroviral vector, generating pMSCVpuro-his-*Rv0927c*.

HEK293T cells were seeded in 10 cm dishes at a density of 2 × 10^6^ cells per dish and incubated for 16 h. To produce retroviruses, cells were co-transfected with pMSCVpuro-*Rv0927c* or pMSCVpuro and pcl-Ampho using Lipofectamine^®^ 3000. Culture supernatants were collected at 48 and 72 h, centrifuged and filtered through a 0.45 µm filter to remove debris, then stored at -80°C until use.

### Generation of stable cell lines

A549 cells were seeded in six-well culture plates at 1 × 10^5^ cells per well. After incubation for 16 h, cell supernatants were removed and replaced with a medium containing viral supernatants and polybrene at a final concentration of 10 µg/mL. To facilitate infection, the plate was centrifuged at room temperature for 90 min. Subsequently, the medium was removed and fresh complete medium was added. A cell line stably expressing Rv0927c (A549-Rv0927c) was selected using puromycin at its minimum effective concentration. Expression was then further validated with Western blot.

### Xenograft tumor model

Six-week-old female nude BALB/c mice were purchased from the Comparative Medical Center of Yangzhou University (Yangzhou, China) and maintained under specific pathogen-free conditions in mouse isolators (Suzhou Monkey Animal Experiment Equipment Technology, Suzhou, China). All animal experiments were approved by the Animal Welfare and Ethics Committee of Yangzhou University and complied with guidelines from the Institutional Administrative Committee and Ethics Committee of Laboratory Animals (IACUC license number: SJXY-7).

After subcutaneous injection of A549-Rv0927c cells (6 × 10^6^) in 100 μL PBS, tumor growth was monitored every 3 days until day 45. When mice were euthanized, tumors were harvested, weighed, and photographed.

### Annexin V/PI assays

Apoptosis was determined using an Annexin V-Alexa Fluor 647/PI Apoptosis Detection Kit (FcMACS). A549 cells and A549 cells pre-transfected with siRNAs were transfected with pCMV-Myc or pCMV-Myc-*Rv0927c* using Lipofectamine^®^ 3000, harvested, and resuspended in 1 × binding buffer (0.1 mL) at a concentration of 1 × 10^6^ cells/mL. Next, cells were stained for 15 min with 5 μL of Annexin V-Alexa Fluor 647 and 10 μL of propidium iodide (PI) at room temperature in the dark. Apoptotic cells were quantified using a FACSCalibur flow cytometer (BD Biosciences).

### Immunoblotting

After transfection with pCMV-Myc and pCMV-Myc-*Rv0927c*, A549 cells were placed in iced lysis buffer containing 100 M phenylmethylsulfonyl fluoride (PMSF) for 30 min. Prestained protein ladders (26617, Thermo Scientific, Waltham, MA, USA) and lysates were separated using SDS-PAGE and transferred to PVDF membranes. Membranes were blocked with 5% skim milk for 1 h at room temperature, then incubated (4°C) overnight with primary antibodies. After immunoblotting with secondary antibodies, images were visualized with an ECL chemiluminescence substrate (Thermo Scientific) in the Amersham Imager 600 Imaging System (GE Healthcare Life Sciences, Pittsburgh, PA, USA).

Primary antibodies were as follows: anti-Caspase-3 antibody (Ac030, Beyotime, Haimen, China), anti-Caspase-9 antibody (9508T, Cell Signaling Technology, Danvers, MA, USA), anti-Caspase-8 antibody (9746T, Cell Signaling Technology), anti-PARP antibody (9542T, Cell Signaling Technology), anti-β-actin antibody (A5441, SigmaAldrich, St. Louis, MO, USA). Secondary antibodies were goat anti-mouse IgG-HRP (401215, Sigma-Aldrich) and goat anti-rabbit IgG-HRP (ab6721, Abcam, Cambridge, MA, USA).

### Mitochondrial membrane potential

The mitochondrial membrane potential (ΔΨm) of A549 cells was measured using a mitochondrial membrane potential detection kit (JC-1, Beyotime). Cells pre-transfected with pCMV-Myc and pCMV-Myc-*Rv0927c* were stained with JC-1 staining solution for 20 min in the dark at 37°C, then washed twice with the provided buffer. Red and green fluorescence indicated positive and negative membrane potential, respectively. Fluorescence was detected using a fluorescence microscope (DMI3000, Leica, Germany).

### Construction of pCMV-HA-TUFM

Human *TUFM* was synthesized by Sangon Biotech (Sangon, Shanghai, China) and cloned into the pCMV-HA vector to generate pCMV-HA-*TUFM*. These plasmids were transformed into *Escherichia coli* DH5α cells via standard heat-shock procedures. TUFM expression was validated using immunoblotting.

### Co-immunoprecipitation assay

Following transfection with pCMV-HA-*TUFM* and pCMV-Myc-*Rv0927c*, HEK293T cells were collected and lysed in western and immunoprecipitation (IP) buffer for 30 min at 4 °C. Lysates were pre-incubated with protein A agarose beads (Cell Signaling Technology) to remove non-specific adsorption, followed by overnight incubation with Myc tag antibody at 4°C. Next, protein A agarose beads were added for a 3 h incubation at 4°C. Subsequently, beads were washed extensively with the western and IP buffer, then resuspended in 50 μL 1 × SDS-PAGE loading buffer. Immunoprecipitated samples were analyzed using immunobloting with HA tag antibody.

### Statistical analysis

All analyses were performed in Prism version 6.01 (GraphPad; GraphPad Software, San Diego, USA). Data are presented as means ± SEM. Between-group differences were assessed with two-tailed unpaired *t*-tests. Significance was set at *p* < 0.01.

## Results

### Rv0927c inhibits lung epithelial cell proliferation

**Figure 1:**
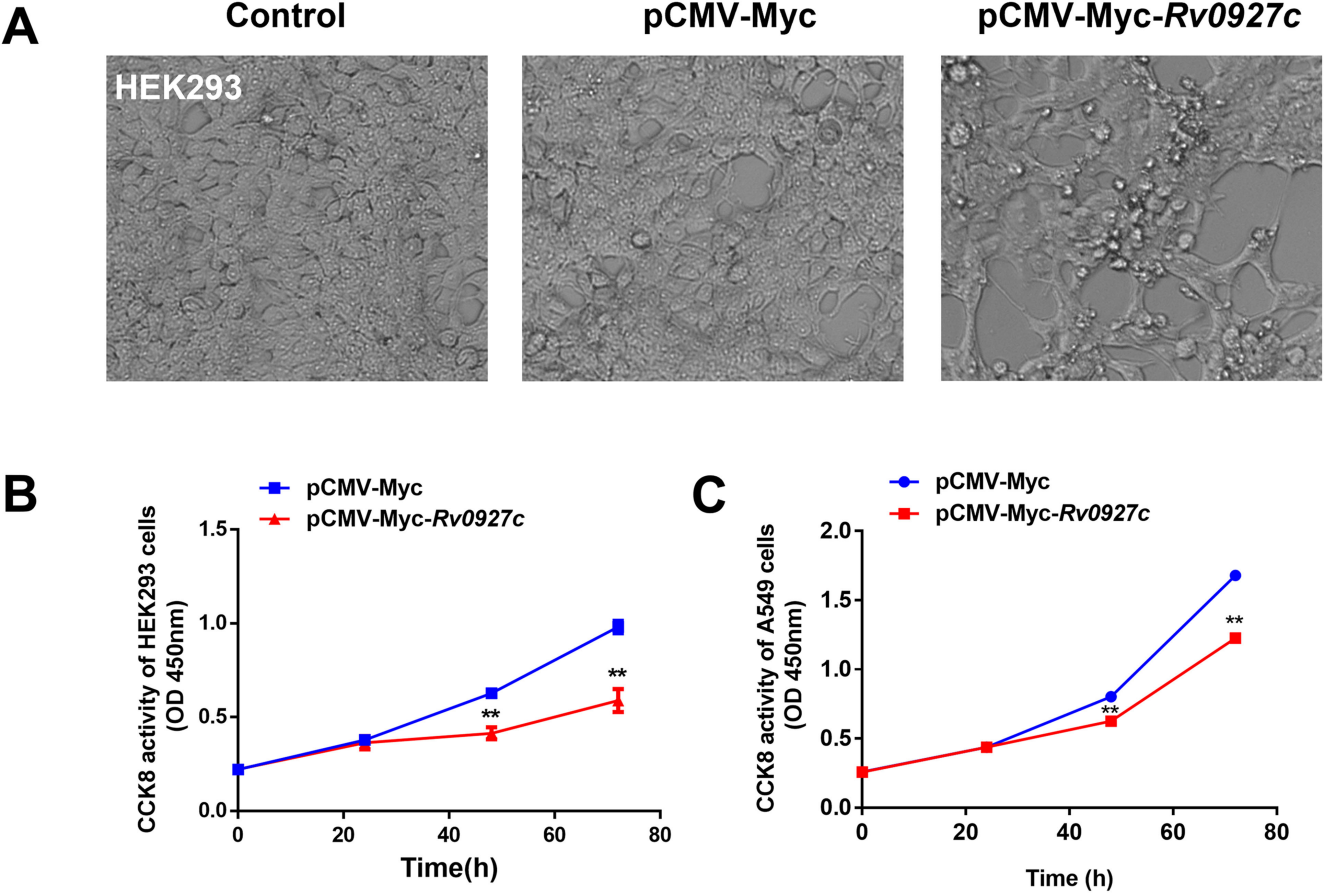
Rv0927c inhibits cell proliferation. **(A)** HEK293T cells were transfected with pCMV-Myc and pCMV-Myc-*Rv0927c*, respectively. Cell morphology was observed under an inverted light microscope. Following transfection with the eukaryotic plasmids pCMV-Myc and pCMV-Myc-*Rv0927c*, HEK293T **(B)** and A549 **(C)** cell proliferation were determined with CCK8 assays. Data shown represent the mean ± SEM of three independent experiments. **P < 0.01.

Transfection with pCMV-Myc-*Rv0927c* led to morphological changes including shrinkage, rounding, and enlargement of cellular gaps (**Figure 1A**). To investigate whether Rv0927c influences cell proliferation, we transfected the eukaryotic recombinant plasmid pCMV-Myc and pCMV-Myc*-Rv0927c* into HEK293T cells and utilized the Cell Counting Kit-8 to determine cell proliferation at various time points. HEK293T cells transfected with pCMV-Myc*-Rv0927c* had lower proliferation rates than those transfected with pCMV-Myc at 24, 48 and 72 h (**Figure 1B**). Consistent with this finding, Rv0927c also inhibits the proliferation of lung epithelial (A549) cells, which are commonly used for studying cellular activity (**Figure 1C**).

**Figure 2:**
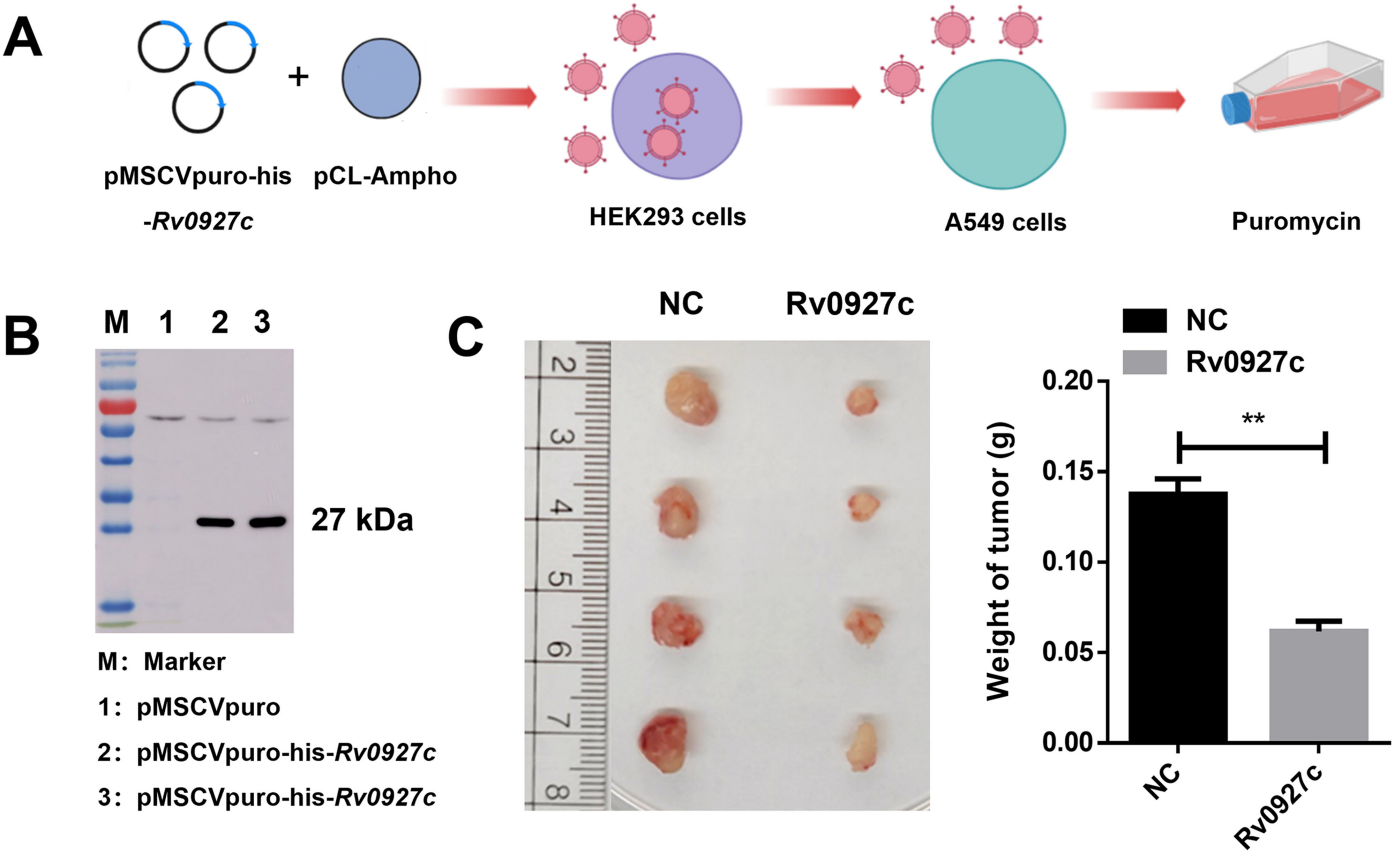
Tumor formation in nude mice. **(A)** Flow chart of pMSCVpuro-his-*Rv0927c* A549 stable cell line construction. **(B)** A549-Rv0927c cells were lysed and analyzed using Western blot with his antibody. **(C)** Tumors in nude mice after subcutaneous injection of A549-Rv0927c cells. Tumors were harvested, weighed, and photographed at 45 days after injection. Results are representative of three independent experiments (mean ± SEM). **P < 0.01.

Furthermore, the effect was further studied *in vivo* using xenograft tumor models. Initially, the recombinant plasmid pMSCVpuro-*Rv0927c* was constructed, and both the retroviral expression vector pMSCVpuro-his-*Rv0927c* and the packaging vector pcl-Ampho were co-transfected into HKE293 cells. The cell supernatants were collected after 48 and 72 hours, and the virus was harvested through centrifugation and filtration (**Figure 2A**). The virus was subsequently used to infect A549 cells with puromycin selection. Western blot analysis confirmed that we had successfully generated a stable A549 cell line expressing Rv0927c (A549-Rv0927c) (**Figure 2B**). A total of 6×10^6^ A549-Rv0927c cells in 100 μL PBS was subcutaneously inoculated into nude mice and tumor growth was monitored. The results showed that the weight of tumors formed by A549-Rv0927c cells were significantly lower than tumor weight in the control group (**Figure 2C**). Collectively, these findings indicate that Rv0927c suppresses lung epithelial cell proliferation.

### Rv0927c promotes apoptosis of lung epithelial cells

**Figure 3:**
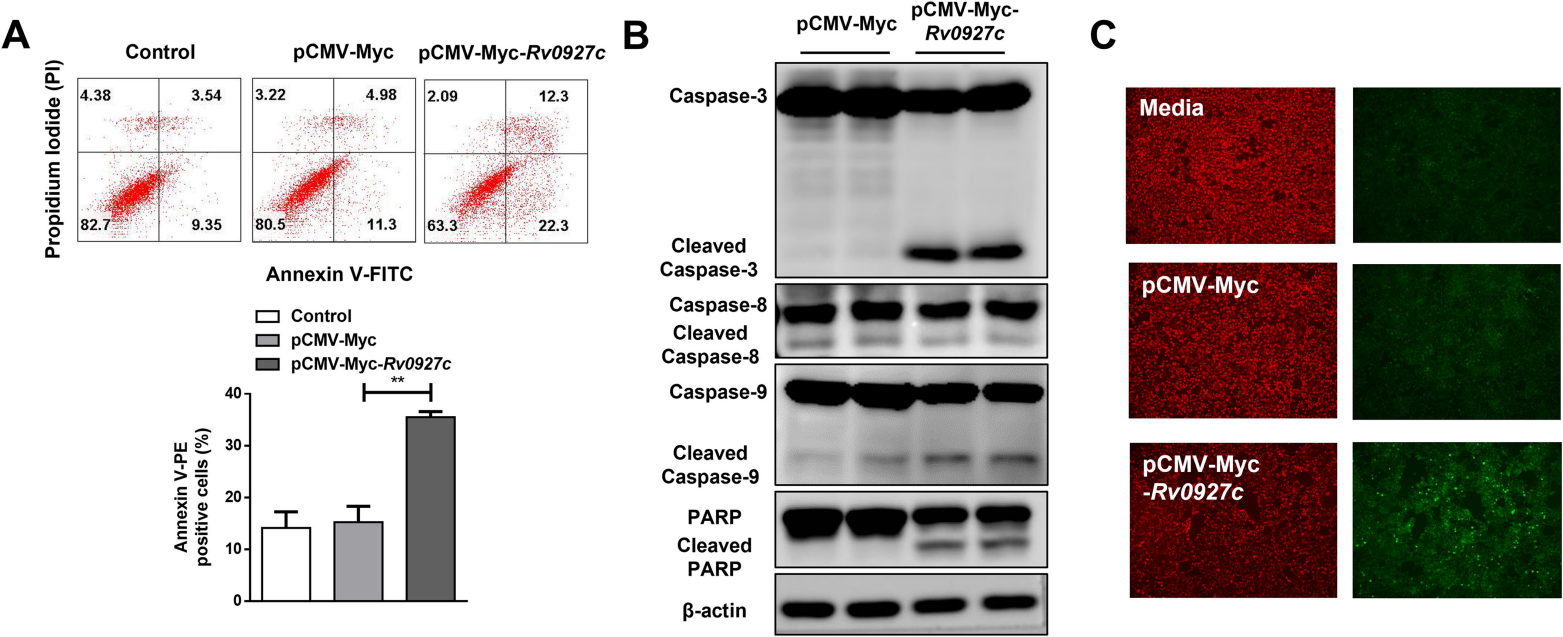
Rv0927c regulates mitochondrial-mediated endogenous apoptotic pathway in lung epithelial cells. **(A)** After transfecting cells with pCMV-Myc and pCMV-Myc-*Rv0927c* for 24 h, cells were stained with PI and Annexin V, followed by flow cytometry analysis (statistical analysis, bottom). **(B)** A fluorescence microscope was used to observe the changes of mitochondrial membrane potential in A549 cells transfected with pCMV-Myc and pCMV-Myc-*Rv0927c*. **(C)** Following transfection with pCMV-Myc and pCMV-Myc-*Rv0927c*, the cells were collected and the levels of cleaved caspase-3, cleaved caspase-8, cleaved caspase-9, and cleaved caspase-parp protein were determined by Western blot. Data shown represent the mean ± SEM of three independent experiments. **P < 0.01.

Apoptosis, also known as programmed cell death, is the primary mechanism for preventing tumor growth (29). Therefore, we performed flow cytometry analysis to examine the apoptotic levels of untreated A549 cells or transfected with pCMV-Myc/pCMV-Myc-*Rv0927c* for 24 h. As shown in **Figure 3A**, it was found that the proportion of the total (AnnexinV^+^), early (AnnexinV^+^/PI^−^) and late (AnnexinV^+^/PI^+^) apoptosis cells in the untreated control group and pCMV-Myc transfected group was 12.89%, 9.35%, 3.54% and 16.28%, 11.3%, 4.98%, respectively. In contrast, following transfection with pCMV-Myc-*Rv0927c*, the proportions of the total, early and late apoptotic cells increased to 34.6%, 22.3% and 12.3%, indicating that overexpression of Rv0927c enhanced the apoptosis of lung epithelial cell.

### Rv0927c induces apoptosis through the endogenous apoptotic pathway

Apoptosis can be triggered by two pathways: the endogenous pathway mediated by caspase-9 and the exogenous pathway mediated by caspase-8 (30). These two pathways ultimately converge on the activation of caspase-3, which leads to apoptotic DNA fragmentation through cleavage of multiple target proteins (31). To investigate the pathway through which Rv0927c induces apoptosis, we determined the expression levels of apoptosis-related proteins using Western blot analysis. The results indicated that Rv0927c promoted caspase-9 cleavage but did not affect caspase-8 cleavage. Correspondingly, the levels of cleaved caspase-3, which serves as a key executor of apoptosis, and cleaved PARP, a cleavage substrate of caspase-3, are also increased in pCMV-Myc-*Rv0927c*-transfected cells compared to those transfected with pCMV-Myc (**Figure 3B**). Furthermore, JC-1 staining assay was performed to determine whether Rv0927c-induced apoptosis was related to the mitochondrial apoptosis pathway. In the untreated and pCMV-Myc transfected cells, the JC-1 probe aggregates within the mitochondrial matrix, forming J-aggregates that display red fluorescence, which is indicative of a high mitochondrial membrane potential. However, J monomers are formed in pCMV-Myc-*Rv0927c* transfected cells, resulting in a shift from red to green fluorescence (**Figure 3C**), indicating that Rv0927c induces a decrease in mitochondrial membrane potential. These results suggested that Rv0927c induces apoptosis via the mitochondrial-mediated endogenous apoptotic pathway.

### Rv0927c interacts with mitochondrial TUFM

**Figure 4:**
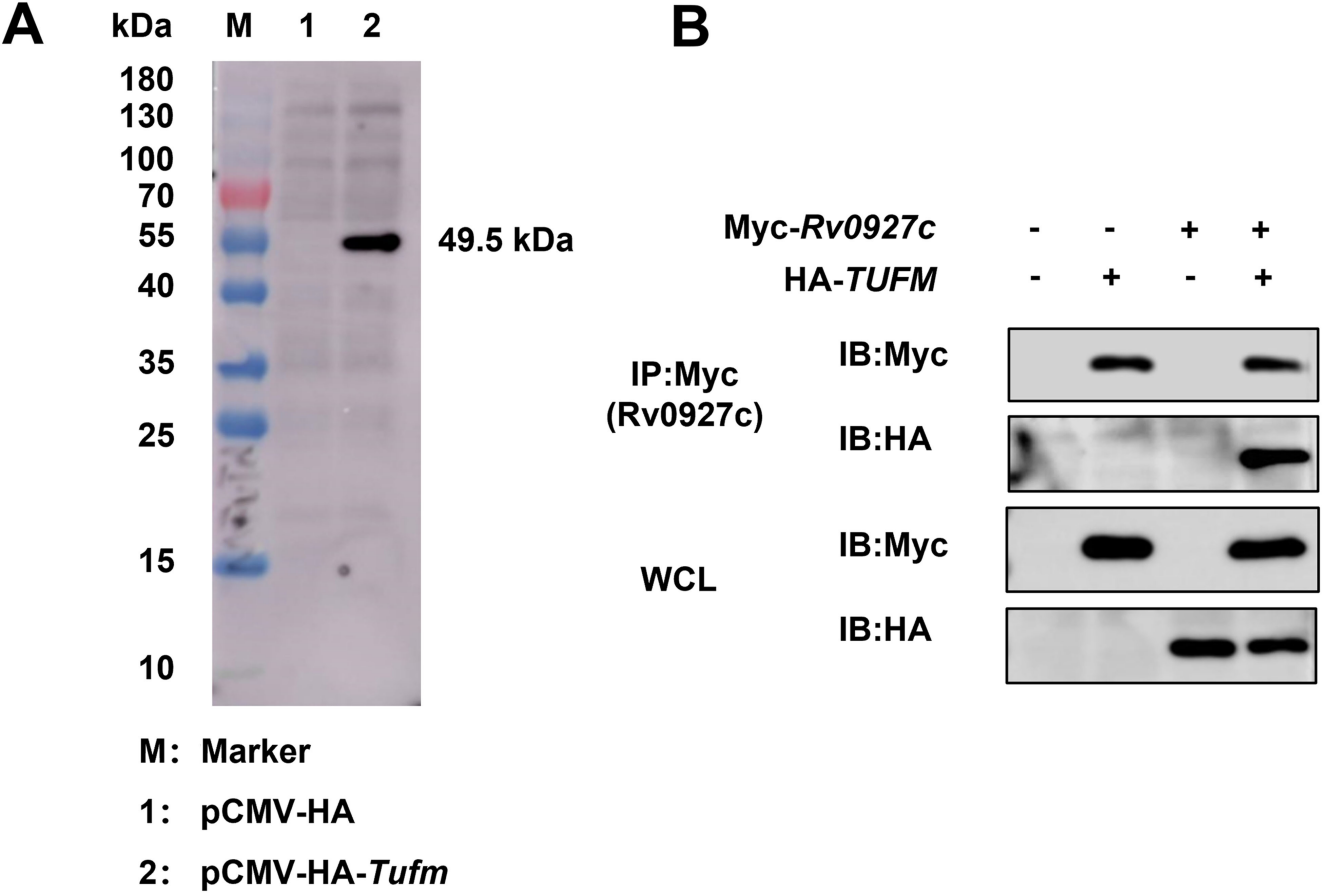
Rv0927c interacts with TUFM. **(A)** Whole cell lysates of HEK293T cells transfected with pCMV-HA (Lane 1) or pCMV-HA-*TUFM* (Lane 2) were collected and the expression of TUFM was determined by Western blot using HA antibody. **(B)** Recombinant plasmids pCMV-Myc-*Rv0927c* and pCMV-HA-*TUFM* were transfected into HEK293T cells and the interaction of Rv0927c with TUFM was confirmed by Co-IP assay.

To further investigate the underlying mechanism by which Rv0927c promotes lung epithelial cell apoptosis, we identified crucial host factors involved in this process. In our previous study, yeast two-hybrid (Y2H) assay was carried out to identify the probable Rv0927c-interacting proteins, one of these cDNA sequences in the prey plasmid was found to have very high similarity with mitochondrial Tu translation elongation factor (TUFM). TUFM protein plays a crucial role in the translation of mitochondrial proteins and is closely associated with the activity of the mitochondrial respiratory chain (32). To verify the interaction between Rv0927c and human TUFM, we constructed the recombinant plasmid pCMV-HA-*TUFM*. The recombinant plasmid was then transfected into HEK293T cells, and the whole cell lysate was collected and analyzed by Western blot. A single specific band, of 49.5 kDa, was observed (**Figure 4A**), confirming the successful expression of the eukaryotic plasmid, which can be utilized for co-immunoprecipitation. The eukaryotic plasmid pCMV-Myc-*Rv0927c* and the pCMV-HA-*TUFM* were co-transfected to HEK293T cells, with anti-Myc antibodies employed for immunoprecipitation. We confirmed that Rv0927c interacts with TUFM (**Figure 4B**).

### Rv0927c regulate lung epithelial cells apoptosis in a TUFM-dependent manner

**Figure 5:**
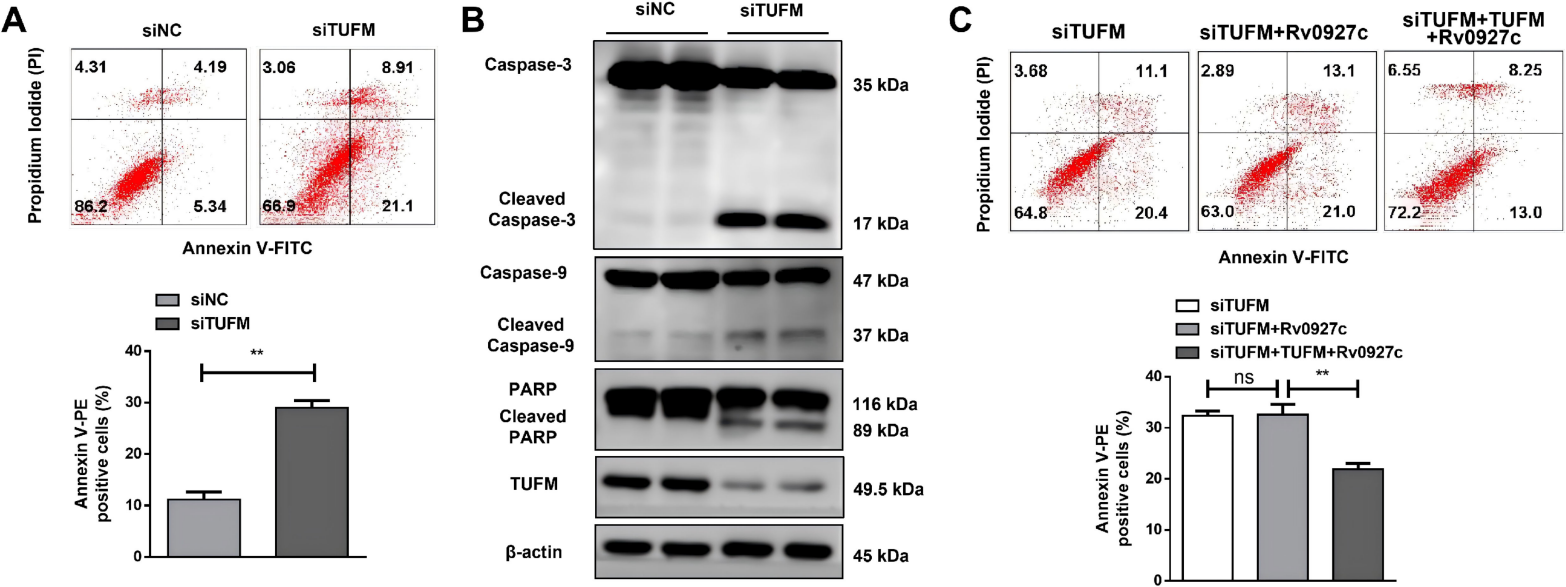
Rv0927c regulates apoptosis in lung epithelial cells via TUFM. TUFM siRNA was used to silence TUFM expression in A549 cells. **(A)** Apoptotic cells were determined by Annexin V/PI staining assays (statistical analysis, bottom). **(B)** The expression levels of apoptosis-related proteins were determined by Western blot. **(C)** The levels of apoptosis in A549 cells that were pre-transfected with siRNA and then transfected with pCMV-Myc-*Rv0927c* or together with pCMV-HA-*TUFM* were analyzed using flow cytometry. Data shown represent the mean ± SEM of three independent experiments. **P < 0.01.

To explore the potential role of TUFM in regulating cell apoptosis, siRNA was used to silence the expression of TUFM, with unrelated gene siRNA (siRNA-NC) as a control. Cells was collected 72 h post-transfection, and apoptosis levels was determined by flow cytometry. Cells transfected with siRNA-TUFM showed much high total (35.3% vs 16.71%), early (24.1% vs 12.3%) and (11.2% vs 4.41%) apoptotic rations than those transfect with siRNA-NC (**Figure 5A**). Simultaneously, Western blot analysis was employed to assess the protein levels of apoptosis-related molecules. The results indicated that the silencing of TUFM enhanced the cleavage of Caspase-9, Caspase-3, and PARP (**Figure 5B**), suggesting that TUFM plays a negative role in regulating the endogenous apoptosis of host cells. To explore whether TUFM is involved in regulating the cell apoptosis induced by Rv0927c, A549 cells were pre-transfected with siRNA and then transfected with pCMV-Myc-*Rv0927c* or together with pCMV-HA-*TUFM*. The results showed that knockdown of TUFM expression abolished the pro-apoptotic effect of Rv0927c, as apoptosis levels were comparable between the siTUFM group and the siTUFM plus Rv0927c group. Further, re-expression of TUFM significantly attenuated Rv0927c-induced apoptosis (**Figure 5C**), indicating that Rv0927c-induced apoptosis is dependent on TUFM.

## Discussion

*M. tuberculosis* is a highly successful intracellular pathogen that has co-evolved with its host to employ diverse strategies for immune evasion and persistence (33). Alveolar epithelial cells, particularly type II cells, play an important role in tuberculosis infection by serving as an alternative replication niche, secreting cytokines and contributing to barrier integrity (5–9). Manipulation of epithelial cell fate by *M. tuberculosis* proteins may facilitate bacterial dissemination and progressive tissue pathology (34). In our previous study, Rv0927c was shown to suppress proinflammatory cytokine production in macrophages by inhibiting NF-κB activation and p38 signaling, consequently promoting intracellular mycobacterial survival (28). The current work reveals a distinct function of Rv0927c in alveolar epithelial cells, which inhibits the proliferation of A549 cells and suppresses tumor formation in mice, thereby laying the groundwork for further investigation into the relationship between *M. tuberculosis* infection and alveolar epithelial cells.

Interestingly, while the present study demonstrates that Rv0927c acts as a proliferation-inhibiting factor in alveolar epithelial cells, earlier reports have identified other *M. tuberculosis* proteins with apparently opposing effects on the same cell type. PtpA enters the host cell nucleus, directly binds to the promoter of GADD45A via its N-terminal DNA-binding region, suppresses its transcription, and thereby promotes A549 cell proliferation (19). Similarly, Mce2E promotes proliferation of A549 epithelial cells by inhibiting K48-linked polyubiquitination and proteasomal degradation of eEF1A1 (20). These functional differences likely reflect the sophisticated, stage-specific strategies of *M. tuberculosis* to balance host cell survival and death during infection. PtpA and Mce2E may help establish an early replicative niche in epithelial cells, whereas Rv0927c may impair barrier function and tissue repair at later stages.

Apoptosis is triggered by multiple signaling pathways and is regulated by both extrinsic and intrinsic ligands (30). The signals associated with apoptosis primarily focus on the activation of pro-caspases, which are central to the initiation and execution of this process (35). Caspases, a family of proteases, play a crucial role in programmed cell death and inflammation by indiscriminately degrading proteins (36). In exogenous apoptosis, caspase-8 is activated through cell death receptors such as FAS/TNF-α (37). In contrast, during endogenous apoptosis, caspase-9 and caspase-3 are subsequently activated by the mitochondrial damage (38). Mitochondria are recognized as the targets for manipulation by various bacteria and viruses, with these pathogens hijacking mitochondrial functions to determine the fate of infected cells (39). Mitochondria play multiple roles in the activation of endogenous apoptosis, including the release of cytochrome C, alterations in electron transport, reductions in mitochondrial membrane potential, and changes in cellular redox status (40). Apoptotic proteins that target mitochondria can induce mitochondrial swelling or the leakage of apoptotic effectors by increasing mitochondrial membrane permeability (41). Among mycobacterial proteins, ESAT-6-mediated apoptosis represents an intrinsic pathway that activates caspase-9 and caspase-3 through the mitochondrial release of cytochrome C (42). In this study, Rv0927c is shown to induce apoptosis via the mitochondrial-mediated endogenous apoptotic pathway.

To identify the probable Rv0927c interacting proteins, one of these cDNA sequences was found to be mitochondrial Tu translation elongation factor (TUFM) with yeast two-hybrid (Y2H) screening in our previous study (unpublished data). The interaction between R0927c and the human TUFM protein was confirmed through co-immunoprecipitation. *TUFM* gene encodes the mitochondrial elongation factor EF-Tu, a highly conserved GTPase. The GTPase and tRNA binding activity of TUFM facilitates amino acid elongation by promoting the delivery of aminoacyl-tRNAs to the A site of the mitochondrial ribosome (43, 44). Some studies have shown that TUFM is associated with cell apoptosis. Choi et al. showed that TUFM acts as an inhibitor of altered mitochondria-induced apoptosis through its autophagic function (45). Zhong et al. indicated that reducing the expression of TUFM with siRNA promoted early apoptosis and the protein levels of Bax and cleaved caspase-3 were increased (46). Interestingly, cell viability was significantly reduced in the cells transfected with TUFM siRNA (46). In this study, we demonstrated that TUFM was negatively correlated with apoptosis. Moreover, Rv0927c overexpression did not significantly induce apoptosis in the TUFM-knockdown group. These outcomes suggest that Rv0927c targets TUFM to induce apoptosis in lung epithelial cells.

Although Rv0927c was predicted to be predominantly membrane-associated, our previous experiments demonstrated that Rv0927c localizes to both the cytosolic and cell wall fractions within mycobacteria (28). More importantly, when host cells were infected with mycobacteria, Western blot analysis of isolated host cytosolic fractions revealed detectable Rv0927c protein in the host cytosol (unpublished data). This provides direct evidence that Rv0927c is released or secreted into the host cytosol during infection, enabling potential interaction with mitochondrial TUFM. In addition, proteomic analyses of *M. tuberculosis* culture filtrate have shown that a substantial number of proteins annotated as cell wall, membrane or lipoprotein-associated are detectable in the culture supernatant, indicating that many predicted cell envelope proteins are in fact exported or released into the extracellular milieu (47–49).

Majority of our experiments relied on ectopic overexpression of Rv0927c to investigate its direct effects on alveolar epithelial cells. Overexpression systems are widely used to dissect the specific mechanistic contributions of individual bacterial effectors (50, 51), as they allow precise control of protein levels and facilitate the study of direct host-pathogen interactions in isolation from other bacterial factors. However, this approach bypasses the natural secretion and localization constraints imposed by *M. tuberculosis*. Consequently, the levels and timing of Rv0927c delivery in our system may differ from those occurring during actual infection. Future studies using Rv0927c-deficient or complemented *M. tuberculosis* strains will be essential to confirm the physiological relevance of these findings.

In conclusion, our results indicate that Rv0927c inhibits A549 cells proliferation and tumor formation. Furthermore, Rv0927c promotes apoptosis via the endogenous pathway dependent on host TUFM. Based on previous and current findings, we propose a unifying model in which Rv0927c acts as a context-dependent putative virulence factor. In macrophages, it suppresses inflammatory responses to enhance mycobacterial survival. In alveolar epithelial cells, it may impair barrier function and tissue repair through TUFM-dependent apoptosis, potentially promoting bacterial release from apoptotic cells. Although direct evidence for bacterial dissemination derives from analogous mechanisms of other *M. tuberculosis* virulence factors (22, 52), this coordinated manipulation could ultimately contribute to chronic infection, tissue damage, and cavity formation.

## Data Availability

The datasets presented in this study can be found in online repositories. The data that support the findings of this study are openly available in “Mendeley Date” at https://doi.org/10.17632/4yczxggxxh.2.
